# Idiopathic granulomatous mastitis after ductoscopy: A case report

**DOI:** 10.1016/j.ijscr.2021.106540

**Published:** 2021-11-02

**Authors:** S. Makineli, P.J. van Diest, M.A. Fernandez, A.J. Witkamp

**Affiliations:** a Department of Surgical Oncology, University Medical Center, Utrecht, the Netherlands; b Department of Pathology, University Medical Center, Utrecht, the Netherlands; c Department of Radiology, University Medical Center, Utrecht, the Netherlands

**Keywords:** Breast disease, Ductoscopy, Granulomatous mastitis, Nipple discharge

## Abstract

**Introduction and importance:**

Idiopathic granulomatous mastitis (IGM) is an uncommon, benign, chronic inflammatory breast disease of unknown etiology, unpredictable duration, and unclear therapy.

**Presentation of case:**

A 41-year-old woman presented with pathological nipple discharge for which ductoscopy was performed. Post-ductoscopy, the patient developed abscesses in her breast with histopathological confirmation of granulomatous mastitis (GM).

**Clinical discussion and conclusion:**

IGM has an unknown etiology and atypical presentation. This is the only case described in which IGM occurred after ductoscopy. This can be related to trauma-induced GM or underlying IGM aggravated by ductoscopy.

## Highlights

• Idiopathic granulomatous mastitis (IGM) has an atypical presentation.
• Radiological findings are non-specific in diagnosing IGM.
• GM can occur after ductoscopy. Whether this is trauma-induced GM or underlying IGM aggravated by ductoscopy remains unknown.

## 1 Introduction

Idiopathic granulomatous mastitis (IGM) is an uncommon, benign, chronic inflammatory breast disease of unknown etiology and unpredictable duration. Symptoms of IGM are a mass, pain, erythema, swelling, fistula, areolar retraction, ulceration, and abscesses [1], [2]. The rarity of IGM causes a lack of data. Therefore, the best therapy remains unclear.

We describe a case of a woman who developed abscesses after ductoscopy, diagnosed as IGM on biopsy. To the best of our knowledge, IGM after ductoscopy has not been previously described. This report was written in line with the SCARE criteria [3].

## 2 Presentation of case

**Fig. 1:**
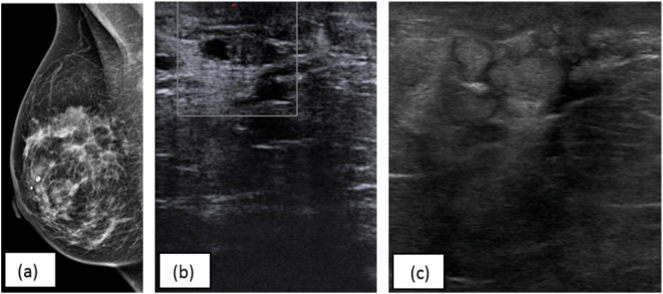
(a) Mammography suggests a retroareolar cyst (b) Ultrasound before ductoscopy: dilated retroareolar ducts (c) Ultrasound after ductoscopy: duct ectasia, not suspicious for an infection or malignancy.

A 41-year-old gravida 3, para 3 woman presented to the outpatient clinic with a 2-year history of right-sided pathological nipple discharge (PND) with unremarkable medical history and drug history. She had milky/brown nipple discharge, which started days before her menstruation and persisted during her menstruation. Physical examination of the breast was normal. Mammography suggested a retroareolar cyst in the right breast (shown in Fig. 1a). Ultrasound showed dilated retroareolar ducts, some solid without vascularization (shown in Fig. 1b). Needle biopsy of this solid part and cytology of nipple discharge showed no abnormalities. We decided to perform a ductoscopy to diagnose and treat possible intraductal lesion(s). The ductoscopy procedure was performed by an experienced breast surgeon. Two major ducts were explored. They were filled with yellow debris, which was collected for pathology. Ductoscopy showed no lesions suspect for malignancy, infection, or benign lesions. After the procedure, the patient went home in good condition.

**Fig. 2:**
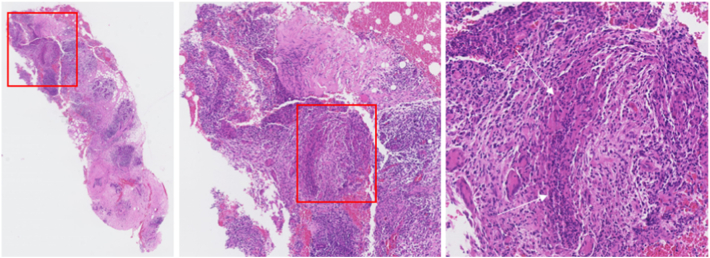
Core needle biopsy showing GM with extensive infiltration of the breast parenchyma by epithelioid histiocytes, multinucleated giant cells and lymphocytes/(H&E).

Four days after ductoscopy, she developed pain and a palpable mass of 4–5 cm in the lower-outer quadrant. Ultrasound showed duct ectasia, not suspicious for infection or malignancy (shown in Fig. 1c). Histological analysis of the debris from ductoscopy showed no abnormalities. Antibiotics were started, and the patient initially reacted well but presented ten days later with abscesses. Surgical incision and drainage were performed. One week after surgery, she slowly recovered. A few days later, a new red palpable mass developed in the outer upper quadrant of the breast. An ultrasound-guided biopsy was performed. Histopathology of this biopsy showed granulomatous mastitis (shown in Fig. 2), for which the patient was referred to an infectious diseases specialist. There was no evidence of other underlying or related illnesses, and it was decided to watch-and-wait. In the follow-up period (one year), she recovered slowly from the remaining abscesses as a self-limiting process.

### 2.1 Retrospective analysis of radiological images

A breast radiologist (AF) retrospectively analyzed the ultrasounds and mammography. The mammography showed more asymmetry in the retromamillar region right than left. The ultrasound images before ductoscopy showed duct ectasia, some with thickened duct wall. This can be related to granulomatous mastitis, but it was very limited in these images and not immediately suspect. The ultrasounds images after ductoscopy showed again duct ectasia, now filled with echogenic material. Therefore, the thickening of the wall was less clear. Because there was no vascularization, the images were not immediately suspect for granulomatous mastitis.

## 3 Discussion

IGM is an uncommon benign chronic inflammatory breast disease first described by Kessler and Wolloch in 1972 [4]. Although the pathogenesis of IGM remains unclear, factors that have been related to the disease include a reaction to trauma, autoimmunity, hormonal or metabolic processes, and infection with *Corynebacterium Kroppenstedtii* [2].

This case study is the first to report the unusual presentation and complicated diagnosis of a patient with IGM after ductoscopy. In our case, we hypothesize that the patient may have suffered from PND, with GM as an underlying cause, and the ductoscopy procedure aggravated the chronic inflammatory disease, which led to the formation of abscesses in the breast. Another hypothesis is that the patient was suffering from PND from another cause, and the ductoscopy was a traumatic event that led to “trauma-induced GM”. In three previously reported cases, a possible “trauma-induced GM” was also described: in one case induced by a foreign body [5] and in two cases by a blunt trauma[6], [7].

Ductoscopy is a minimally invasive procedure with mild and rare complications. Only mild nipple pain, bacterial mastitis, and perforation of the ductal lining have been reported [8]. No other case of IGM after ductoscopy has been described before.

The diagnosis and treatment of IGM are challenging because of a lack of valid data. Currently, the application of corticosteroids and the use of surgery in cases with insufficient response to conservative treatment is the most common approach [1], [2], [9]. In this presented case, IGM was treated by surgery without steroids. The patient developed abscesses, but these were self-limiting. The treatment was difficult and prolonged. Therefore, good communication about disease management is crucial.

## 4 Conclusion

IGM has an unknown etiology and atypical presentation. Radiological findings are non-specific in diagnosing IGM. This is the only case described in which IGM occurred after ductoscopy. Whether this concerns trauma-induced GM or underlying IGM aggravated by ductoscopy remains unknown. With this case report, we want to create awareness of the rare presentation of this condition after ductoscopy.

## Guarantor

S. Makineli and A.J. Witkamp

## Consent for publication

Written informed consent was obtained from the patient for publication of this case report and accompanying images.

## Sources of funding

None declared.

## Ethical committee approval

Ethics approval was not required for case reports according to guidelines in the Netherlands.

## Provenance and peer review

Not commissioned, externally peer-reviewed.

## CRediT authorship contribution statement

S. Makineli: conceptualization, methodology, project administration, resources, writing – original draft.

P.J. van Diest: methodology, supervision, validation, writing – review and editing.

M.A. Fernandez: writing – original draft

A.J. Witkamp: conceptualization, methodology, supervision, validation, writing – review and editing.

## Declaration of competing interest

The authors declare that they have no conflict of interest.
